# Genetic Behavior of Tomato (*Solanum lycopersicum* L.) Germplasm Governing Heavy Metal Tolerance and Yield Traits under Wastewater Irrigation

**DOI:** 10.3390/plants11212973

**Published:** 2022-11-03

**Authors:** Shameem Raja, Fozia Farhat, Arneeb Tariq, Zaffar Malik, Rana Badar Aziz, Muhamamd Kamran, Mohsen Mohamed Elsharkawy, Asif Ali, Abdulrahman Al-Hashimi, Mohamed S. Elshikh

**Affiliations:** 1 Department of Botany, Faculty of Science and Technology, Government College Women University, Faisalabad 38000, Pakistan; 2 Department of Soil Science, Faculty of Agriculture & Environmental Sciences, The Islamia University of Bahawalpur, Bahawalpur 63100, Pakistan; 3 Department of Horticulture, Nanjing Agricultural University, Nanjing 210095, China; 4 School of Agriculture, Food and Wine, The University of Adelaide, Adelaide 5005, Australia; 5 Department of Agricultural Botany, Faculty of Agriculture, Kafrelsheikh University, Kafr El-Sheikh 33516, Egypt; 6 Department of Plant Breeding and Genetics, Muhammad Nawaz Sharif University of Agriculture, Multan 66000, Pakistan; 7 Department of Botany and Microbiology, College of Science, King Saud University, Riyadh 11451, Saudi Arabia

**Keywords:** North Carolina Mating II, gene action, dominance, additive

## Abstract

Wastewater irrigation is a substitute for surface water scarcity, but traces of heavy metals (HMs) result in deleterious implications for soil, crop productivity, and in humans. Crops presenting HMs tolerance in genetic behavior are important for producing tolerant genotypes cultivated under wastewater irrigation. In the first part of this experiment, the results obtained previously are re-assessed in a hydroponic system and similar patterns and concentrations of HMs are found in different tomato organs. Following this trial, the tomato’s (*Solanum lycopersicum* L.) genetic basis of traits conferring HMs tolerance and yield are assessed when irrigated with waste or canal water. The North Carolina Mating II analysis illustrate the amount of gene action, nature, and inheritance pattern. Genetic components depict the involvement of non-additive, additive, and maternal genetic effects in HMs tolerance inheritance and yield. A noticeable increase in cumulative additive variance for the number of flowers (11,907.2) and the number of fruits (10,557.9) is recorded for tomato plants irrigated with wastewater, illustrating additive gene action. However, female and male (MSf/MSm) square ratios also show an association with cytoplasmic inheritance. For HMs tolerance, both additive and dominant variances appeared to be significant; cumulative dominance variance (4.83, 16.1, 4.69, 76.95, and 249.37) is higher compared to additive variance (0.18, 2.36, 0.19, −0.27, and 14.14) for nickel (Ni), chromium (Cr), lead (Pb), manganese (Mn), and zinc (Zn), respectively, indicating dominance gene action. The genotype RIOGRANDI accumulated and translocated fewer HMs to the aerial part of the plant compared to CLN-2418A and PB-017906, thus presenting a tolerant tomato genotype according to the hydroponic experiment. This also exhibited a differential pattern of gene action for HMs tolerance, suggesting that genotypes possess significant differences for HMs tolerance.

## 1. Introduction

The agriculture sector alone accounts for utilizing more than two-thirds of the total water around the globe. However, semiarid regions are at a high risk to ongoing water shortage situations [1]. Furthermore, with a rapidly growing population, water demand and wastewater discharge raise the probability of wastewater reuse [2]. On the other hand, wastewater irrigation can enhance electrical conductivity, total dissolved solids, major ion concentrations, exchangeable sodium concentration, and exchangeable sodium cation (Na^+^) percentage (ESP) [3].

Wastewater is a major source of environmental pollution that moves to higher organisms through the food chain [4,5,6]. A significant proportion of people live in comparatively smaller communities located in distant areas, where domestic and industrial wastewater is still generally poorly treated or even kept untreated, usually discharged into rivers or streams having high biodiversity values [7]. Different forms of contaminants in the wastewater accumulate in sewage sludge (80–90%), and thus discharge of the sludge into waterbodies would increase organic load tremendously with a concurrent decrease in dissolved oxygen (DO) levels and nutrients [8].

To address the food security and climate issues of the increasing population, one has to re-think non-conventional water resources. This present scenario of limited water resources has stimulated scientists to work on alternative plans for freshwater management and wastewater recycling [9]. Currently, under such circumstances of surface water scarcity, wastewater irrigation is in need of time and is gaining importance due to rapid surface water reduction [2]. On the other hand, untreated or poorly treated wastewater contains many HMs, causing soil and consequently plant pollution, which has increased many times in recent years, and in some parts of the world, it reaches beyond the permissible limit for all living things [10]. Heavy metals and pathogens in wastewater pose potential threats to soil, plants, and humans [11]. A major part of Pakistan is currently irrigated with wastewater (32,500 ha) [12] and a significant fraction of this wastewater either poorly treated or non-treated is utilized for crop irrigation. At a national level, ~1% of industrial and >10% of municipal wastewater gets treatment [13]. The reuse of treated municipal wastewater, however, is an alternate source of irrigation to address water scarcity issues [14].

The quality of irrigation water accessible to the local farmers and others has a potential effect on the plants/plant species that can be cultivated, the yield of the plants, water infiltration, and other soil physio-biochemical properties. Irrigation via sewage water is the basic cause of the deposition of HMs in vegetables [15]. On the other hand, if such practices keep going at such a pace, they can induce changes in the quality of soil and available water over a long period [16]. The sewage water is being used for the irrigation of edible plants and it is a matter of great concern due to the presence of pollutants, particularly, toxic metals [17].

Tomato (*Solanum lycopersicum* L.) is one of the important food crops worldwide, and due to its availability, both as a fresh and processed fruit, it is also commercially important all over the world [18,19]. In Pakistan, the tomato was grown on 60.5 thousand hectares area during 2016–2017, and during the last decade, its production was 569.0 thousand tons with an average yield of 9.4 tons per hectare [20]. Currently, Pakistan exports tomatoes to Sri Lanka, Afghanistan, the U.A.E., Iran, Saudi Arabia, and India [19]. Wastewater irrigation of agricultural lands around urban areas of the whole country is a usual practice due to water shortage and farmers irrigate their crops mostly with wastewater [21]. In this respect, germplasm genotypic screening against HM tolerance and high yield is a good way for HM-tolerant crop genotype selection [22]. Plant yield and tolerance to HM are quantitatively controlled traits and exhibit complex inheritance due to the involvement of genetic and environmental factors [23]. Inheritance of desired characteristics is crucial for breeding, therefore HM tolerance inheritance pattern identification is important for tomatoes in selection and breeding to increase the yield [24]. Tomato crops possess a wide range of genetic heritable variability for different characteristics [25]. Heterosis confers greater vigor, resistance to biotic and abiotic stresses, higher productivity, faster development, and earliness [26]. A plant’s HM tolerance (resistance to metals accumulation) with sustainable yield is controlled by many major and modifier genes [10]. In wheat, Mn tolerance is controlled by a few genes, while in alfalfa, it is controlled by several genes’ additive effects. Likewise, in lettuce, Mn tolerance is attributed to one to four genes, while in soybean it is due to multiple genes [27]. Besides this, cell walls, vacuoles, as well as plasma membranes, all play important roles in HM tolerance [28]. Plant breeding provides important information about the type of gene action, i.e., additive, dominant, or epistasis. Several biometrical techniques, i.e., North Carolina Model, generation mean analysis, diallel and line × tester, are available for genetic analysis. North Carolina Model II is an important biometrical technique to study gene action for a breeding program. 

An insight into the genetics of HM tolerance may allow the development of tomatoes with good tolerance potential against HMs. HM tolerance facilitates plant growth even under higher metal concentrations [29]. In Pakistan, tomatoes are mostly grown in peri-urban areas and irrigated with untreated wastewater due to surface water unavailability [30]. Wastewater irrigation has gained importance due to rapid surface water reduction.

The objective of the experiment was to determine the genetic control of yield and HM tolerance under wastewater irrigation. The genetic component studies, inheritance pattern, and involvement of non-additive, additive, and maternal genetic effects about HM tolerance and yield were observed under both water treatments. Along with this, the study was conducted to find out the involvement of some extra-chromosomal inheritance (maternal effects) to improve yield and HM tolerance in different tomato genotypes. The obtained results will provide a source of potential genetic resources and inheritance patterns, which may be further studied to develop morphological and physio-biochemical markers. 

## 2. Results

### 2.1. Heavy Metals Accumulation under Hydroponic Conditions

**Figure 1:**
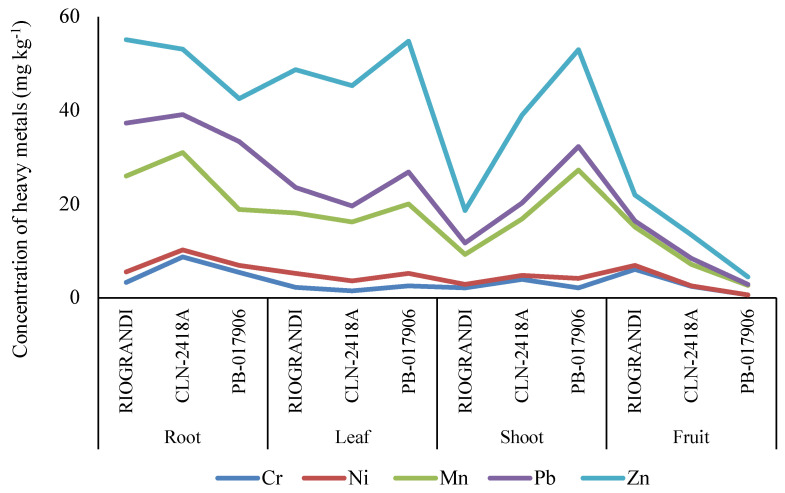
Heavy metals (HMs) accumulation in tomato fruit under the controlled environmental conditions. Abbreviations: Cr: chromium, Ni: nickel, Mn: manganese, Zn: zinc, Pb: lead.

The hydroponic experiment further validated the field trial’s experimental results by showing similar heavy metal accumulation patterns in various parts of the tomato plant. The fruit of genotype PB-017906 showed little concentrations of Ni, Cr, Pb, Mn, and Zn (0.02, 0.58, 0.12, 2.10, and 1.63 mg kg^−1^, respectively), and to some extent, in CLN-2418A (0.08, 2.40, 1.30, 4.58, and 5.02 mg kg^−1^, respectively). In contrast, the fruit of RIOGRANDI exhibited a higher accumulation of these metals (0.89, 5.99, 1.27, 8.20, and 5.58 mg kg^−1^, respectively). According to the data, PB-017906 showed a higher accumulation of HMs, particularly zinc (Zn), in the leaf and shoot parts of the tomato plants, thus being considered as an HM sensitive genotype, while RIOGRANDI is an HM tolerant genotype based on low HM accumulation (Figure 1). However, RIOGRANDI shows a higher accumulation of HMs in roots than other genotypes, which may have restricted further translocation to the aerial parts of the tomato plants. In contrast to the varying HM accumulation in fruit, other plant parts similar HM accumulation patterns were found in other plant parts of observed genotypes (Figure 1). Moreover, sensitive genotypes noted fewer flowers and fruits under HMs stress conditions.

### 2.2. Variations in Yield and Heavy Metals Accumulation

The data were analyzed by analysis of variance (ANOVA), and genotypic differences were non-significant for all traits, while male, female, and male × female interactions were significant under two treatments. However, significant differences were observed among genotypes for yield-related traits and the number of flowers and fruits (Table 1). Female and male (MSf/MSm) square ratios were significant for the number of flowers and fruits under both treatments (wastewater and canal water irrigation. Genotypic differences for heavy metals tolerance were found to be significant (Table 1).

### 2.3. Tomato Yield-Related Traits

Yield-related traits’ gene action can be assessed through simple genetic models. Still, due to the polygenic nature of the total number of fruits, interaction effects disturb the Mendelian ratio and lead to undesirable phenotypes. σ^2^_m_ = male additive variance (601.3), and σ^2^_f_ = female additive variance (8329.1) was higher than σ^2^_m×f_ = m × f interaction additive variance (268.9). However, higher values of cumulative additive variance (11,907.2) than dominance variance (1075.4) reveal that an additive type of gene action controls this characteristic. Meanwhile, the degree of dominance was less than 1 (i.e., 0.30), which showed a partial type of dominance for the number of fruits (Table 2). A similar pattern of gene action was observed for the number of flowers, depicting significant genotypic differences (Table 2). σ^2^_f_ = Female additive variance (7576.3) was higher than σ^2^_m_ = male additive variance (342.2) and σ^2^_m×f_ = m × f interaction additive variance (745.5). Higher cumulative additive variance than dominance variance reveals an additive type of gene action. The degree of dominance was 0.53, which showed partial dominance under wastewater irrigation (Table 2). Present studies showed additive and non-additive gene actions and maternal effects were involved for yield-related traits and in tomatoes, depicting cytoplasm involvement. Higher values of additive variance and lower values of degree of dominance [σ^2^_H_/σ^2^_D_]^1/2^ showed partial dominance for the number of flowers and fruits and confirmed the additive type of gene action. Along with additive and non-additive genetic effects, maternal effects were also involved in the number of flowers and fruits (Table 2).

Under canal water irrigation, a similar gene action pattern was observed for the total number of flowers and fruits (Table 2). Female additive variances (6639.95) were higher as compared to male additive variances (424.15) and male × female interaction (474.31) for the total number of fruits. Similarly, female additive variances were higher for the total number of flowers (7300.9) as compared to male additive variances (377) and male × female interaction (551.3). These findings depict higher cumulative additive variance than dominance variance for both traits, which shows additive gene action. The degree of dominance was lower than 1, and partial dominance was observed for both characteristics (Table 2). 

Heritability is considered an excellent index for the transmission of characteristics to subsequent generations for yield contributing traits improvement. The heritability of yield-related traits was 0.99, while genetic advance was between 111.8 and 106.3 for the number of fruits and flowers under wastewater application. Under canal water irrigation, broad sense heritability and genetic advance for the number of fruits and flowers were observed to be 0.91, 0.99, and 106, respectively (Table 3).

### 2.4. Heavy Metals Tolerance

A differential pattern of gene action was found for HM tolerance compared to yield-related traits, suggesting that genotypes possess significant differences for HM tolerance (Table 2). σ^2^_f_ = female additive variance was higher than σ^2^_m_ = male additive variance, but σ^2^_m×f_ = m × f interaction additive variance was higher than either male or female additive variance. However, the values of dominance variance (4.83, 16.1, 4.69, 76.95, and 249.37) were higher than cumulative additive variance (0.18, 2.36, 0.19, −0.27, and 14.14) for Ni, Cr, Pb, Mn, and Zn, respectively (Table 2). Dominance variance was higher than the additive variance for HMs tolerance. The degree of dominance for Ni, Cr, Pb, Mn, and Zn uptake was 5.1, 2.5, 4.9, −16.7, and 4.2 under wastewater irrigation. A higher degree of dominance >1 revealed the presence of overdominance for metals tolerance. Higher female than male additive variance indicates that female metal tolerance improvement will be effective in subsequent generations because selection will fix the additive portion. A higher degree of dominance [σ^2^_H_/σ^2^_D_]^1/2^ showed over-dominance for HMs tolerance, which indicates that HMs tolerance is controlled by both additive and non-additive genetic effects (Table 2).

Heritability and genetic advance estimates also provide information about HM tolerance inheritance patterns. Traits possessing higher genetic advances with high or low heritability estimates are desirable because of their higher additive genetic effects; therefore, selection may be effective based on genetic advances. In the present studies, the broad sense heritability of Cr, Mn, Ni, Pb, and Zn were 0.80, 0.70, 0.73, 0.62, and 0.85, respectively, under wastewater application, while genetic advance was 113.3, 65.1, 91, 73.8, and 36.1 for Cr, Mn, Ni, Pb, and Zn, respectively (Table 3).

Under canal water irrigation, analyses indicated that σ^2^_f_ = female additive variance was higher than σ^2^_m_ = male additive variance. However, σ^2^_m×f_ = m × f interaction additive variance was higher than either male or female additive variance for HMs. Higher dominance variances (1.44, 5.81, 1.66, 15.8, 100.73) were found as compared to cumulative additive variance (0.10, 0.54, 0.23, 0.12, and 6.67) for Ni, Cr, Pb, Mn, and Zn, respectively (Table 2). Table 2 depicted that dominance variance was higher than the cumulative additive variance for all HMs tolerance. The degree of dominance for Ni, Cr, Pb, Zn, and Mn uptake was 3.8, 3.3, 2.7, 11.5, and 3.9 under canal water irrigation. A higher degree of dominance than one revealed over-dominance for all metals tolerance. Both female and male MSf/MSm square ratios were significant for Ni, Cr, Pb, Mn, and Zn tolerance under both treatments (wastewater and canal water), indicating extra-chromosomal inheritance involvement for HMs tolerance (Table 2). However, this inheritance was not included in the female additive variance. A similar gene action pattern was observed for HM tolerance under canal water irrigation. Significant differences were observed in HM tolerance among genotypes (Table 1). When canal water was used for irrigation, the broad sense heritability was 0.51, 0.72, 0.60, 0.69, and 0.82, and the genetic advance was 77.7, 98.5, 79.9, 81.9, and 41.8 for Ni, Cr, Pb, Zn, and Mn, respectively (Table 3).

## 3. Discussion

During the initial tomato germplasm screening, the soil-grown plants in an open field trial were affected by soil, manure, animals, birds, insects, and humans, transmitting the pathogens before or during the harvesting [31]. Plants grown without soil using hydroponic technology in greenhouses within a closed environment could be at lower interference with other physical and external variables, making the results more reliable. The toxicity of metals in hydroponic systems has been evaluated earlier by Cd, Cu [32], Ni, and Se [33], demonstrating the adsorption of these elements and the phytoremediation efficiency in ornamental and medicinal crops. Mexican mint in hydroponic systems was found to accumulate Pb in the roots without translocating it to stems or leaves [34]. While water spinach uptakes Pb from water through roots and translocates it to stems and leaves, with the greatest accumulation in the root [35]. The process is completed with a biological component that includes intracellular intake, vascular deposition, and sprout translocation to the shoots. Our results follow these earlier reported findings, as the leaf, root, stem, and fruit parts of the tomato exhibited the same pattern of HM accumulation as were found in the field experiment (Figure 1). This requires a mechanism of surface sorption involving chelation, ion exchange, and specific absorption [36].

Genetic variation plays an important role in developing potential plant material for improved yield and HMs tolerance. For the efficient use of this variation, the desired characters should be genetically controlled [37]. Selection of breeding procedures adopted to improve desired traits such as HMs tolerance and yield is possible if knowledge of genetic effects governing the inheritance is available. The inheritance of desirable characteristics is crucial for any breeding program. Therefore, studying the inheritance pattern for tolerance to HMs is important for selection and breeding. Information about the genetic basis (additive and non-additive) of the desired trait can help the breeder select the most effective breeding method and the best accessions [38]. In the current trial, chromosomal additive and non-additive inheritance transmission patterns were involved. Evaluation of studied yield and HM accumulation attributes can be assessed through simple genetic models but due to the involvement of multiple genes in the expression of traits, the Mendelian ratio is affected. North Carolina Mating Design II (NCM II) also provides precise information not only about chromosomal but also extra-chromosomal (maternal effects) inheritance.

In addition, many female and male parents can be used in the crossing plan, although the main drawback of this design is that it cannot provide any information about epitasis effects. To analyze the genetic behavior (additive and non-additive) of the desired traits in tomatoes, NCM II was used [39]. The inheritance model of tomato yield attributes is complicated and involves polygenic interactions [40]. Genetic effects and inheritance patterns for yield and HMs tolerance were determined. In tomatoes, the number of flowers per branch, the number of fruits per branch, and fruit weight are all major yield-contributing traits controlled by multiple genes [19]. 

The results have shown that additive and nonadditive gene actions and maternal effects were taken part in controlling different traits of yield and HMs tolerance in tomatoes, indicating the involvement of cytoplasm. Higher values of additive variance for the number of flowers and fruits showed that an additive type of gene action controlled these traits; therefore, selection would be effective in subsequent generations [36,41]. For this breeding program, recurrent selection would be required [42]. Higher values of additive variance and lower values (less than 1) of a degree of dominance [σ^2^_H_/σ^2^_D_]^1/2^ showed partial dominance for the number of flowers and the number of fruits and confirmed that an additive type of gene action controlled these characteristics. Along with additive and non-additive gene effects, maternal effects were also involved in controlling the number of flowers, the number of fruits, and HMs tolerance, indicating the interference of the cytoplasm. HM Involvement of maternal effects for multiple HM tolerance through the MTP gene family has also previously been reported by El-Sappah et al. [43]. Mitochondria, chloroplast, and plastid DNA are involved in the inheritance of traits, along with nuclear genes [4]. Cell walls, vacuoles, and plasma membranes play important roles in HM tolerance [44]. This study demonstrated the involvement of some extra-chromosomal inheritance (maternal effects) in HMs tolerance and yield-related traits.

Heritability is considered an excellent index for transmitting characteristics to subsequent generations. To realize improvement in yield-contributing traits, a plant breeder must assess the percentage of inheritance in a population for any particular trait. It also enlightens the efficiency of subsequent generations regarding the selected traits in any breeding experiment. Genotypic, as well as environmental variance, can be assumed to approximate the chances of heritability and genetic advance, which depicts the probability of genetypic differences concerning environmental variables. Heritability and genetic advance were high with high additive gene effects for the number of flowers and fruits; therefore, direct selection without hybridization may help improve these characteristics [45]. These results suggest that both additive and dominant gene effects were significant for tomato dominance variance, which was higher than the additive variance for HMs tolerance [46]. Therefore, hybrid development may be effective for metals tolerance using males and females in the next generations, and progeny testing may be necessary for hybrid development [47]. Metal tolerance was controlled by dominant gene action, indicating that hybrid production is the best way to use the existing variability for this trait [39]. Higher values of female than male additive variance indicate that improving metals tolerance selection for females will be effective in subsequent generations because the additive portion can be fixed through selection. The higher values of degree of dominance [σ^2^_H_/σ^2^_D_]^1/2^ showed over-dominance for the control of HMs tolerance, indicating that both additive and non-additive genetic effects control HMs tolerance.

Heritability and genetic advance estimates also provide information about yield and HM tolerance inheritance patterns. Traits possessing higher genetic advances with high or low heritability estimates are desirable because of their higher additive genetic effects; therefore, selection may be effective based on genetic advances. Higher heritability estimates with lower genetic advances are undesirable because of non-additive genetic effects, which show that environmental effects regulate trait expression. Heritability and genetic advance were high to moderate for Pb, Cr, Mn, and Ni tolerance but not Zn. Therefore, direct selection without hybridization may be helpful for these characteristics [48]. Because genetic advance is the direct measure of additive variance, but the accumulative additive variance was less than the dominance variance for HM tolerance, high heritability, and genetic advance for Pb, Cr, Mn, and Ni may be due to the female plants, which possess higher values of additive variance [49]. 

Estimates for different genetic components revealed that an additive genetic effect was responsible for the inheritance of yield-related traits such as the number of flowers and the number of fruits per plant. In contrast, a dominant genetic effect was responsible for the inheritance of different HMs tolerance. From the above results, it was observed that along with additive and non-additive genetic effects, maternal effects were involved in controlling different traits such as yield and HMs tolerance in tomatoes, showing the involvement of cytoplasm. An evaluation of the many hybrids/accessions is recommended to develop low metal-accumulator tomato genotypes with a higher yield. Because the above results are from a limited set of tomato germplasm, they cannot be generalized to all tomato genotypes [50]. 

Multiple genes influenced genetic variation for metal tolerance with both additive and non-additive effects, but for HM tolerance, dominant genetic effects were observed most commonly [51], which are quite helpful for producing the best hybrid genotypes. In tomatoes, extra-chromosomal patterns also participate in inheriting some traits [52], including HM tolerance. Such maternal effects are inherited through genes located in plasmids. In contrast, yield-related traits were mainly determined by additive genetic effects [53]. Maternal effects were observed for the inheritance of the number of flowers and number of fruits in tomatoes [54]. 

## 4. Materials and Methods

### 4.1. Experiment

#### 4.1.1. Re-Assessment of Initial Screening Experiment

Two HM tolerant/low metal-accumulator genotypes (PB-017906 and CLN-2418A) and one susceptible/high metal accumulator genotype (RIOGRANDI) were selected out of 44 genotypes in a screening trial based on HM accumulation in a field experiment (Shamim et al. 2022) and then results were re-assessed in the hydroponic experiment. A similar pattern of HM concentrations “as in sewage water” (Table 1) was maintained under hydroponic conditions supplemented with Hoagland nutrient solution. Ni, Pb, Mn, Zn, and Cr concentrations were assessed in different plant organs of tomatoes under hydroponic conditions by atomic absorption spectrophotometer. The 0.1 g of each dried plant part (root, shoot, leaves, and flower) was digested in HNO_3_ for 3 h under lab conditions. The samples were heated at 250 °C on a heating block until the appearance of tinted yellow color of the solution in digestion flask. The solution was diluted with distilled water up to 50 mL and filtered for the analysis of HMs with atomic absorption spectrophotometer. 

#### 4.1.2. Breeding Experiment

Seeds of the selected tomato genotypes were sown in sand-filled seedling trays, and moisture content was maintained. After 25 days of seedling establishment, in November 2018, seedlings were transplanted to the field soil in a replicated trial under two treatments, i.e., wastewater and canal water. At maturity, the crossing was performed according to North Carolina Design-II matting to develop genetic material for assessing genetic components of variability. Analysis of water and soil used in current experiment given in Table 4.

### 4.2. Emasculation

Floral buds of all genotypes in both water treatments were covered with a butter paper bag and emasculated with forceps two days before blooming. Any opened flowers present on the same inflorescence were removed to avoid mating. 

### 4.3. Pollen Collection

Desired male flowers of all the genotypes in both water treatments were stored in clean plastic bottles, and anthers were collected and sun-dried, so they opened to release pollens for crossing. 

### 4.4. Pollination

Pollens were applied to exposed stigma with a dissecting needle; to ensure a good seed set, the style was repeatedly dipped in a pollen-containing petri dish.

### 4.5. Seed Extraction

At maturity, fruits of F1 and selected parents were collected in bags, crushed, and fermented. Seeds were washed with fresh water to remove flesh and skin. Floating pieces were removed gently, and washing was repeated many times by adding fresh water to remove flesh and gel. Clean seeds were collected, dried, and stored. 

### 4.6. Assessment of Plant Material for Genetic Studies

To assess inheritance patterns and genetic studies for HMs tolerance and yield-related traits, HMs tolerant (PB-017906 and CLN-2418A), susceptible parental genotypes (RIOGRANDI), and F_1_ hybrids seeds were sown in seedling trays and transplanted in field after 25 days of the seedling establishment during November 2019 in a triplicated trial in two sets, i.e., canal water and wastewater. Data regarding HM and yield-related traits were recorded at maturity by harvesting and analyzing ripened fruit. Accumulation of nickel (Ni), zinc (Zn), manganese (Mn), chromium (Cr), and lead (Pb) in tomato fruit were determined by performing acid digestion as described by [55]. For yield assessment, mean values for the number of flowers per truss and the number of fruits in each cluster were recorded for each genotype.

### 4.7. Statistical Analysis

Data were analyzed statistically using the analysis of variance technique [56] to find significant genotypic differences among parental and hybrid genotypes for HM tolerance and yield potential. Genetic components of variation, grand mean, phenotypic and genotypic variances were computed [57]. Broad sense heritability (h_BS_) and genetic advance (GA) were calculated using formulas [58,59]. Values for genetic advance have been categorized as low (10%), medium (up to 20%), and high (more than 20%). The below 30% heritability estimate was designated as low, in the range of 30–60%, indicating medium heritability, and above 60% estimates were considered high [60]. Data were subjected to North Carolina Mating design-II analysis to determine gene action for various traits [61].

## 5. Conclusions

Under such circumstances of surface water scarcity, wastewater is used for irrigation purposes, but one of its drawbacks is contamination with HMs. Due to the high cost, treatment is not possible in underdeveloped countries such as Pakistan. One of the solutions to this problem is to develop genetically tolerant genotypes that hold genetic capability physiologically and possess molecular mechanisms to block HMs from entering the food chain. Genotypic variation exhibited a differential pattern of gene action for HMs tolerance, suggesting that genotypes possess significant differences for HMs tolerance. Since roots are the first organ for the hyperaccumulation of different HMs ions as shown in different tomato genotypes, particularly RIOGRANDI, therefore, the pattern of enzyme activities and other metal accumulation in the root system needs to be determined, and intermittent use of clean water between wastewater irrigation will not only reduce the heavy metal load but could also enhance the soil fertility. 

## Figures and Tables

**Table 1:** Analysis of variance for attributes related to yield and HM accumulation in tomato fruit irrigated with wastewater and canal water (North Carolina matting design-II).

	Source	NOFL	NOF	Cr	Pb	Zn	Ni	Mn
Wastewater	Replication	71 ns	8 ns	0.235 ns	0.004 ns	0.69 ns	0.2448 ns	0.363 ns
	Males	9478 **	13438 **	5.13 **	4.68 **	160.37 **	1.36 **	17.36 **
	Females	206,852 **	225,698 **	73.51 **	8.86 **	549.45 **	11.50 **	118.97 **
	M × F	2293 **	811 **	14.06 **	4.78 **	205.25 **	4.19 **	64.26 **
	Error	56	5	1.98	1.26	18.22	0.57	6.54
	MSf/MSm	21.8	16.8	14.3	1.9	3.4	8.4	6.9
Canal water	Replication	2 ns	27 ns	0.0871 ns	0.0695 ns	0.130 ns	0.2419 ns	0.259 ns
	Males	9601 **	10,383 **	2.6534 **	1.2923 **	29.208 **	1.0211 **	11.652 **
	Females	198,808 **	180,755 **	18.3007 **	6.6098 **	283.268 **	3.6906 **	19.598 **
	M × F	1683 **	1476 **	4.7114 **	1.5910 **	81.290 **	1.3026 **	14.064 **
	Error	29	53	0.3506	0.3447	5.743	0.2240	2.215
	MSf/MSm	20.70	17.41	6.90	5.123	9.69	3.6	1.68

NOFL: number of flowers, NOF: number of fruit, Cr: chromium, Pb: lead, Zn: zinc, Ni: nickel, Mn: manganese, ns: not significant, **: highly significant at 1% MSf/MSm: mean squares female/mean squares male.

**Table 2:** Genetic components of variation for yield and HMs accumulation under wastewater and canal water irrigation (North Carolina matting design-II).

	Genetic Component	NOF	NOFL	Zn	Cr	Mn	Ni	Pb
Wastewater	σ^2^_m_	601.3	342.2	−2.14	−0.42	−2.23	−0.13	−0.01
	σ^2^_f_	8329.1	7576.3	12.75	2.20	2.03	0.27	0.15
	σ^2^_m×f_	268.9	745.5	62.34	4.03	19.24	1.21	1.17
	σ^2^_H_	1075.4	2981.9	249.372	16.1	76.9	4.83	4.69
	σ^2^_D_	11,907.2	10,557.9	14.15	2.37	−0.28	0.18	0.20
	[σ^2^_H_/σ^2^_D_]^1/2^	0.30	0.53	4.19	2.54	−16.69	5.13	4.89
Canal water	σ^2^_m_	424.2	2ns	−2.48	−0.098	−0.115	−0.013	−0.014
	σ^2^_f_	6639.95	9601	7.48	0.50	0.21	0.089	0.186
	σ^2^_m×f_	474.31	198,808	25.18	1.45	3.9	0.36	0.42
	σ^2^_H_	9418.8	1683	6.67	0.54	0.12	0.10	0.23
	σ^2^_D_	1897.22	29	100.73	5.81	15.8	1.44	1.66
	[σ^2^_H_/σ^2^_D_]^1/2^	0.45	20.70	3.88	3.28	11.46	3.79	2.69

NOFL: number of flowers, NOF: number of fruit, Zn: zinc, Cr: chromium, Mn: manganese, Ni: nickel, Pb: lead, σ^2^_m_: male additive variance, σ^2^_f_: female additive variance, σ^2^_m×f_: m × f interaction additive variance, σ^2^_H_: dominance variance, σ^2^_D_: cumulative additive variance, [σ^2^_H_/σ^2^_D_]^1/2^: degree of dominance.

**Table 3:** Genetic components of variation for attributes related to yield and HM accumulation in tomato fruit irrigated with wastewater and canal water.

	Traits	PV	PCV %	GV	GCV %	h^2^bs	GA%	EV	ECV %
Wastewater	NOFL	77	60	77	60	0.9	106	46	4.71
	NOF	88	63	88	63	0.9	111	4	1.45
	Cr	15	75	13	70	0.8	113	2	29
	Ni	1.6	73	1.1	61	0.7	91	0.5	39
	Zn	105	24	90	22	0.8	36	15	9
	Mn	23	50	17	43	0.7	65	6	26
	Pb	2.7	67	1.7	53	0.6	73	1.03	41
Canal water	NOFL	7465	60	7434	60	0.99	106	31	3.9
	NOF	7375	66	6740	63	0.91	107	634	19
	Cr	4	77	3	65	0.7	98	1.3	40
	Ni	0.6	85	0.33	61	0.5	77	0.31	59
	Zn	45	28	37	26	0.8	41	8.1	12
	Mn	9	67	6	56	0.6	81	2.9	37
	Pb	1.01	74	0.61	58	0.6	79	0.39	46

PV: phenotypic variance, PCV%: phenotypic coefficient of variance, GV: genotypic variance, GCV%: genotypic coefficient of variance, h^2^bs: broad sense heritability, GA: genetic advance, EV: environmental variance, ECV%: environmental coefficient of variance.

**Table 4:** HMs mean concentrations in water and soil.

Water Samples	Cr (mg L^−1^)	Ni (mg L^−1^)	Mn (mg L^−1^)	Pb (mg L^−1^)	Zn (mg L^−1^)
Sewage Water	10 ± 0.14	2.5 ± 0.05	1 ± 0.02	1.5 ± 0.01	6.5 ± 0.05
Canal Water	8.5 ± 0.06	2 ± 0.06	6.5 ± 0.05	0 ± 0.00	4.5 ± 0.06
Soil (0–20 cm)	0.41 ± 0.09	0.43 ± 0.02	11.57 ± 0.12	1.16 ± 0.02	1.41 ± 0.02
Soil (20–40 cm)	0.4 ± 0.03	0.26 ± 0.04	5.69 ± 0.04	0.62 ± 0.03	0.43 ± 0.02

Cr: chromium, Ni: nickel, Mn: manganese, Pb: lead, Zn: zinc, mg L^−1^: milligrams per liter.

## Data Availability

The datasets used and/or analyzed during the current study are available from the corresponding author on reasonable request.
